# Study on the characteristics and correlation of fecal microbiota and metabolites in patients with acute lung injury after cardiopulmonary bypass based on 16S rRNA sequencing and non-targeted metabolomics analysis

**DOI:** 10.3389/fimmu.2025.1713650

**Published:** 2026-01-12

**Authors:** Shuyuan Yi, Lan Luo, Ziyuan Dong, Kan Wang, Zicheng Zhu, Qian Gao, Yu Jiang, Xiaofang Yang, Feilong Hei

**Affiliations:** Department of Extracorporeal Circulation and Mechanical Circulation Assistants, Center for Cardiac Intensive Care, Beijing Anzhen Hospital, Capital Medical University, Beijing, China

**Keywords:** 16S rRNA sequencing, acute lung injury, cardiopulmonary bypass, gut microbiota, gut-lung axis, metabolomics

## Abstract

Acute lung injury (ALI) is a severe complication following cardiopulmonary bypass (CPB), associated with high mortality and impaired patient prognosis. At present, there is no effective therapeutic strategy for ALI after CPB. Although the gut microbiota has been implicated in ALI, the biological significance of these associations remains largely elusive. A prospective, single-center, case-control design was adopted. A total of 53 post-CPB patients were enrolled, including 21 in the ALI group and 32 in the non-ALI (NALI) group. Postoperative fecal samples were collected for microbiome and metabolomic analyses, which were subsequently correlated with clinical data. Results revealed that β diversity analysis indicated distinct differences in microbial community structure (Anosim: R = 0.14, *P* = 0.004; Permanova: R^2^ = 0.058, *P* = 0.008). ALI patients exhibited a significant increase in the *Bacillota*, alongside reductions in *Bacteroidota* and *Actinomycetota*. At the genus level, *Streptococcus* and *Enterococcus* were enriched in the ALI group, while *Bacteroides* and *Akkermansia* were diminished. Metabolomics analysis identified 130 differentially expressed metabolites, 109 of which were significantly reduced in the ALI group, primarily involving amino acid metabolic pathways such as phenylalanine, tryptophan, and tyrosine. A random forest model identified genera such as *Bacteroides*, *Corynebacterium*, and *Lactobacillus* as having high predictive value for ALI (AUC > 0.7). Combined microbiota-metabolite analysis revealed significant correlations between specific genera and differentially expressed metabolites, suggesting a potential role for the gut-lung axis in the development of ALI following CPB. Patients with postoperative ALI following CPB exhibit marked gut microbiota structural disruption and metabolic dysfunction, both closely associated with adverse clinical outcomes. Genera such as *Bacteroides* and their associated metabolites may serve as early predictive biomarkers, offering novel therapeutic targets for the prevention and management of ALI.

## 1 Introduction

Cardiopulmonary bypass (CPB) is extensively used in cardiac surgery. However, contact between the CPB circuit and circulating blood inevitably triggers an inflammatory response, increasing the risk of postoperative complications such as lung injury, acute kidney injury (AKI), and hemolysis (1–3). One of its complications, acute lung injury (ALI), significantly increases patient mortality and length of hospital stay. ALI is an adverse pulmonary condition accompanied by an inflammatory response, primarily characterized by refractory hypoxaemia and respiratory distress. The pathogenesis of ALI primarily involves damage to endothelial cells and alveolar epithelial cells, inflammatory responses, and oxidative stress (4). A more severe form of ALI is acute respiratory distress syndrome (ARDS), which may progress to multiple organ failure and carries a high mortality rate (5, 6). The incidence of post-CPB ARDS is only 2% to 3%, but its mortality rate reaches as high as 50%, whereas the mortality rate for mild ALI is merely half that (7). ALI following CPB surgery is particularly severe in infants and young children, with an incidence rate of 15–60% (8). Consequently, post-CPB ALI has emerged as the primary cause of increased mortality following cardiac surgery, imposing a substantial medical and societal burden.

The gut is regarded as one of the body’s largest immune barrier systems, serving as a reservoir for both bacteria and endotoxins. Previous reports indicate that the intestinal organ plays a pivotal role in the development of ALI/ARDS (9). As the human body’s ‘second genome’, the gut microbiota interacts closely with the host immune system through its metabolites, forming a complex regulatory network known as the ‘gut-lung axis’. Recent studies have revealed that gut microbiota metabolites, such as short-chain fatty acids, tryptophan derivatives, and polyamines, can influence pulmonary inflammation by regulating immune cell function and maintaining epithelial barrier integrity (10, 11).

Previous studies have identified gut microbiota dysbiosis and compromised intestinal barrier function in pediatric patients undergoing cardiac surgery with CPB support (12). Research has further revealed a close association between post-CPB ALI in infants and preoperative gut microbiota community composition, demonstrating the predictive role of *Escherichia-Shigella* in ALI development (13). Another study examining patients undergoing cardiac surgery with extracorporeal circulation support identified conditionally pathogenic *Escherichia-Shigella* species—which promote inflammatory responses and exacerbate disease progression—as predominant pathogens in the intestines of postoperative ALI patients (14, 15). These findings suggest a relationship between perioperative gut microbiota imbalance and the development of post-CPB lung injury. Post-CPB serum succinate levels correlate positively with mechanical ventilation duration and negatively with the oxygenation index. This suggests that post-CPB lung injury involves the role of the microbial metabolite succinate within the gut microbiota and its effects on lung injury, alveolar damage, and pulmonary inflammation (16).

However, it remains unclear whether lung injury in cardiac surgery patients is associated with specific alterations in gut microbiota and metabolic products. To date, systematic investigations into the gut microbiome and metabolic characteristics of ALI patients following CPB surgery are lacking. This study employs 16S rRNA sequencing and non-targeted metabolomics to systematically analyze differences in gut microbiota and metabolites among ALI patients following CPB-assisted cardiac surgery. It explores potential mechanisms and biomarkers, providing a theoretical foundation for postoperative ALI interventions. We hypothesize that post-CPB cardiac surgery patients may exhibit distinct microbial community structures and metabolite profiles.

## 2 Materials and methods

### 2.1 Method study design, population and sample collection

We conducted a prospective observational, single-center case-control preliminary study at Beijing Anzhen Hospital, Capital Medical University. This study was approved by the Ethics Committee of Beijing Anzhen Hospital, Capital Medical University (Identification Number: KS2025003), and informed verbal consent was obtained from each enrolled patient. We prospectively recruited all patients aged ≥18 years undergoing cardiopulmonary bypass (CPB) surgery between December 2024 and March 2025. Concurrently, patients were required to meet ASA I-III classification criteria with an anticipated surgical duration of ≥2 hours. Exclusion criteria applied: ① Pre-operative infection or severe pulmonary disease (pneumonia, atelectasis, emphysema, pulmonary heart disease, pleurisy, etc.); ② History of acute lung injury or acute respiratory distress syndrome within 3 months prior to surgery; ③ Pregnant or lactating women; ④ BMI > 40 kg/m²; ⑤Patients with functional or organic bowel disorders (e.g., irritable bowel syndrome, ulcerative colitis); those having received continuous antibiotic therapy exceeding 7 days within the preceding 3 months; ⑥ Participants in other clinical trials within the last 3 months; ⑦ Other major comorbidities: severe systemic diseases such as acute myocardial infarction or chronic renal failure. Patients were enrolled consecutively and met identical inclusion and exclusion criteria. All underwent CPB-assisted cardiac surgery using standardized institutional protocols. Because this was an exploratory cohort and the final sample size was modest, formal matching was not performed. All patients received standardized perioperative antibiotic prophylaxis according to institutional guidelines. None of the enrolled patients received prolonged antibiotics (>24 h), prebiotics, probiotics, or bowel-preparation regimens that would substantially alter gut microbiota. Sedation, analgesia, and vasoactive medications followed uniform ICU protocols. Fecal samples were collected within 24–72 hours post-surgery using single-use sterile rectal swabs. All specimens were immediately stored at -80°C until further analysis.

### 2.2 Clinical data

Clinical baseline data including age, weight, gender, medical history, and prognosis were extracted from medical records. Given that oxygenation index(OI, PaO_2_/FiO_2_) are typically low in cardiac surgery patients, we established diagnostic criteria for ALI by referencing Berlin ARDS standards and OI thresholds: ① Postoperative PaO_2_/FiO_2_ ≤ 200 under standardized ventilator settings (PEEP 5–8 cmH_2_O, FiO_2_ ≥ 0.4),the lowest OI value within 24 hours post-surgery should be used for multiple postoperative OI calculations; ② Failure to extubate within 24 hours post-surgery with continued mechanical ventilation support; ③ Pulmonary artery wedge pressure (PAWP) ≤ 18 mmHg or excluded cardiogenic pulmonary oedema by echocardiography, assessment of left ventricular function, and absence of volume overload. Radiological abnormalities were not included as essential criteria for classification in this study. Record preoperative left ventricular ejection fraction (LVEF), echocardiographic ejection fraction (EF) value, and oxygen saturation (SpO_2_) levels, alongside operative duration, ascending aorta cross-clamp time, cardiopulmonary bypass time, and lowest postoperative 24 hours PaO_2_/FiO_2_. Intensive care unit (ICU) length of stay, postoperative mechanical ventilation duration, and hospital stay were selected as clinical outcome measures.

All data were statistically analyzed using SPSS 25.0 software for clinical characteristics. Quantitative data underwent normality testing via the Shapiro-Wilk test; where normality was satisfied, results were presented as mean ± standard deviation (mean ± SD). Intergroup comparisons employed independent samples t-tests (Levine’s analysis confirmed homogeneity of variances). Non-normally distributed data were expressed as median (interquartile range [IQR]) and analyzed using the U test. Qualitative data were presented as N (proportion), with analysis conducted using chi-square tests, continuity-corrected chi-square tests, or Fisher’s exact tests. Differences were considered statistically significant at P < 0.05.

### 2.3 Gut microbiota profiling

Microbial community genomic DNA was extracted using the HiPure Soil DNA Extraction Kit (Magen, China). DNA quality was assessed by electrophoresis on a 1% agarose gel, and concentration and purity were determined using a NanoDrop 2000 UV-vis spectrophotometer (Thermo Scientific, USA). The V3–V4 hypervariable region of the 16S rRNA gene was amplified with primers 341F (5′-TCCTACGGGNGGCWGCAG-3′) and 806R (5′-GGACTACHVGGGTATCTAAT-3′). Amplification conditions were: 95°C for 5 min; 30 cycles of 95°C for 1 min, 60°C for 1 min, 72°C for 1 min; and a final extension at 72°C for 7 min. The reaction mixture (50 μL) contained 10 μL 5× Q5^®^ Reaction Buffer, 10 μL 5× Q5^®^ High GC Enhancer, 1.5 μL 2.5 mM dNTPs, 1.5 μL each primer (10 μM), 0.2 μL Q5^®^ High-Fidelity DNA Polymerase, and 50 ng template DNA (New England Biolabs, USA). PCR products were quality-checked on a 2% agarose gel, purified with AMPure XP Beads (Beckman, USA), and quantified with Qubit 3.0. Libraries were prepared using the Illumina DNA Prep Kit (Illumina, USA) and qualified with an ABI StepOnePlus Real-Time PCR System. Qualified libraries were sequenced on a Novaseq 6000 using PE250 mode.

### 2.4 Fecal metabolomic analysis

Approximately 100 mg of sample was homogenized in 1 mL of pre-chilled methanol: acetonitrile: water (2:2:1, v/v) using an MP homogenizer (24×2 cycles, 6.0 m/s, 20 seconds, repeated three times). Low-temperature ultrasonication was performed for 30 minutes twice, followed by incubation at -20°C for 60 minutes and centrifugation at 13,000 g for 15 minutes at 4°C. The supernatant (900 μL aliquots) was collected, vacuum-dried, and stored as lyophilized powder at -80°C. For mass spectrometry, samples were resuspended in 100 μL acetonitrile-water (1:1, v/v), vortexed, centrifuged at 14,000 g at 4°C for 15 minutes, and the supernatant was injected for analysis.

Chromatographic separation was performed on a Vanquish UHPLC system (Thermo Scientific) equipped with a HILIC column (25°C) at a flow rate of 0.5 mL/min with an injection volume of 2 μL. Mobile phases consisted of A: water + 25 mM ammonium acetate + 25 mM ammonium hydroxide and B: acetonitrile. The gradient program was: 0–0.5 min, 95% B; 0.5–7 min, 95% to 65% B; 7–8 min, 65% to 40% B; 8–9 min, 40% B; 9–9.1 min, 40% to 95% B; 9.1–12 min, 95% B. Samples were maintained at 4°C in the autosampler and analyzed in random order. Quality control (QC) samples were interspersed to monitor system stability and data reliability.

Mass spectrometry was performed on an Orbitrap Exploris™ 48 mass spectrometer. ESI source conditions were: nebulizer gas 50, auxiliary gas 1 (Gas1) 50, auxiliary gas 2 (Gas2) 2; ion source temperature 350°C; ion spray voltage 3500 V (positive mode) or 2800 V (negative mode); primary mass range 70–1200 Da, resolution 60,000, accumulation time 100 ms. Secondary mass acquisition used a segmented method with a range of 70–1200 Da, resolution 60,000, accumulation time 100 ms, and dynamic exclusion time 4 s.

## 3 Data analysis

### 3.1 Analysis of the microbiota

The number of Effective Tags obtained per sample ranged from 56,928 to 122,569, with an average depth of 96,809 Tags per sample. Rarefaction curves reached clear plateaus, and all samples exceeded the rarefaction threshold of 55,000 reads, ensuring adequate depth for downstream analysis. Clean tags were clustered into operational taxonomic units (OTUs) at ≥97% similarity using the UPARSE algorithm in Usearch (v11.0.667) (17). Chimeras were removed using UCHIME (18). Effective tags were subjected to OTU abundance statistics and analyses. The most abundant tag sequence was selected as the representative sequence for each OTU. Species annotation was performed using the RDP classifier (v2.2) against the SILVA database (version 138.2). Alpha diversity indices (Chao1, Shannon) were calculated in R using the formulae referenced at (https://mothur.org/wiki/calculators/). The ggplot2 package (v3.4.2) in R was employed to plot diversity dilution curves, indicating whether sequencing samples possessed sufficient effective data. Differences in alpha indices between groups were analyzed using the R Vegan package (v2.6-4). The Wilcoxon test assessed the significance of differences in alpha diversity indices between two comparison groups, with a default threshold of *P*-value < 0.05. Beta diversity analysis was based on OTU representative sequences. All Wilcoxon rank-sum tests were two-tailed unless otherwise specified. The GuniFrac package (19) in R was employed to compute the weighted_unifrac distance matrix. The Vegan package (v2.6-4) was used to perform principal coordinates analysis (PCoA) based on weighted unifrac distances, with visualization achieved using the ggplot2 package (v3.4.2) in R. A *post hoc* power evaluation was performed to assess the feasibility of the present exploratory cohort. Using effect sizes reported in prior microbiome studies after major surgery, we estimated that a sample size of 50–60 participants provides >80% power to detect differences in β-diversity of R²≥ 0.10 using PERMANOVA (999 permutations, α = 0.05). The present sample size (n = 53) therefore meets the threshold for exploratory multi-omics analysis. We acknowledge that the sample size is not powered for small effect sizes or extensive multivariable modeling; therefore, all findings should be interpreted as preliminary and hypothesis- generating. Species distribution bar charts were generated at the phylum and family levels, showing the top seven most abundant species; others were aggregated as “Other” or “Unclassified”. Circos plots displayed the top ten genera. Shared and unique species were visualized via Upset plots (20). Wilcoxon tests were performed using the R language Vegan package (v2.6-4). LEfSe software (version 1.0) was employed to screen biomarker species within each group. LEfSe (v1.0) was used to identify biomarker species (LDA score ≥ 2, p*P* < 0.05). Statistical tests utilized box plots to display the top eight differentially abundant microbial species between the two groups at the species level under the Wilcoxon rank-sum test, with a threshold of p-value < 0.05. Species abundance ternary plots were generated using the ggtern package in R. Bacterial microbial phenotypes were classified using BugBase (version 1.0), followed by statistical analysis with the Wilcoxon rank-sum test at a default threshold of p-value < 0.05.

KEGG (Kyoto Encyclopedia of Genes and Genomes) metabolic pathway prediction was performed using PICRUSt2 (version 2.5.3). PICRUSt2 statistical tests employed STAMP analysis results and box plots to reflect the degree of functional differences between the two groups at different classification levels within the KEGG database. If the overall distance between boxes for comparative groups was substantial, the likelihood of significant differences between these groups was higher. The Wilcoxon signed-rank test was used, with a default threshold of *P*-value < 0.05. Subsequently, clinical characteristics of the two patient groups were correlated with microbial distribution. Heatmaps were generated using the Omicsmart dynamic real-time interactive online data analysis platform (http://www.omicsmart.com), with Spearman’s correlation coefficients displayed. The Random Forest algorithm was employed to calculate the contribution of differential microbial communities to ALI occurrence, outputting the Mean Decrease in Accuracy index. Species were then ranked according to their contribution weight.

### 3.2 Metabolomics

Following sample pre-processing, metabolites were detected using an AB SCIEX Triple TOF 6600 mass spectrometer before proceeding to bioinformatics analysis. Detection employed both positive ion mode (POS) and negative ion mode (NEG) ionization techniques. Subsequent data analysis processed data from each ionization mode separately. Principal Component Analysis (PCA) provides an overall representation of the general metabolic differences between groups and the degree of variation within samples. PCA analysis of the metabolome data was performed using the R package gmodels. Furthermore, Partial Least Squares Discriminant Analysis (PLS-DA) pre-groups multidimensional data according to the desired discriminating factors (by presetting Y values for target classification and discrimination) prior to compression, thereby reducing the influence of other factors (21). The R package ropls (v1.30.0) was employed for PLS-DA analysis. To gain deeper insight into the up- or down-regulated changes in differential metabolite abundance, volcano plots were generated. These plots combined the fold change values within the comparison groups with the differential metabolites selected based on variable importance in the projection (VIP) and *P*-values. Within the plots, metabolites closer to either end of the x-axis exhibit greater differential abundance. The x-axis represents the logarithmic value of the fold change between the two groups, while the y-axis displays the -Log10 value of the false discovery rate (FDR) or *P*-value for the group difference. Different colors denote differentially expressed metabolites (up- or down-regulated) filtered by threshold, with blue points indicating no difference. Criteria: fold change (FC) ≥ 1, *P* ≤ 0.05, VIP ≥ 0.

Through OPLS-DA analysis, each metabolite yields a VIP value, or Variable Importance in Projection. A higher VIP value indicates a greater contribution of that substance toward distinguishing between the two groups. Differential metabolites between comparison groups were normalized (z-scores) before undergoing cluster analysis. Heatmaps generated using the R package pheatmap (v1.0.12) visually illustrated accumulation differences of these metabolites across both groups. Following the identification of differential metabolites, metabolic pathway enrichment analysis is performed using KEGG. Enrichment plots are generated with R packages such as ClusterProfiler and Enrichplot to visualize significant bubbles. Employing hypergeometric tests, pathways significantly enriched among differential metabolites relative to the entire metabolome background were identified. Significance bubble plots may be used to describe the proportion and significance of differential metabolites within each pathway category. Based on p-values, bubble plots were generated for the top 20 ranked KEGG pathways.

In order to integrate microbiome and metabolomics data, we subsequently employed the R programming language to calculate Spearman’s correlation coefficients between gut microbiota and metabolites at each taxonomic level. Spearman’s correlation serves to measure the mutual relationship between two variables, representing the strength of their covariation and thereby assessing the association between microbial components and metabolites. To assess whether biomarker selection was consistent across different statistical frameworks, we performed a sensitivity analysis using penalized logistic regression with least absolute shrinkage and selection operator (LASSO) regularization. The optimal λ parameter was selected by 10-fold cross-validation. The taxa identified by random forest (*Bacteroides*, *Corynebacterium*, and *Lactobacillus*) remained among the top-ranking predictors in the LASSO model, supporting their robustness as candidate biomarkers.

## 4 Results

### 4.1 Clinical characteristics of patients participating in the study

This study included 53 patients following CPB surgery. Based on the Berlin criteria and established grouping standards, patients were categorized into ALI group (n=21) and NALI group (n=32). **Table 1** summarizes patient characteristics. No significant differences were observed between the ALI and NALI groups regarding age, gender, body mass index (BMI), hypertension, diabetes mellitus, hyperlipidaemia, cerebrovascular disease, SPO_2_, or left ventricular ejection fraction (LVEF) (*P*>0.05). Regarding surgical factors, there was no significant difference in ascending aorta clamp time between groups. However, significant differences were observed in total operating time (334.21 ± 89.36 vs. 268.52 ± 55.78 hours, *P* = 0.002) and cardiopulmonary bypass (CPB) duration (median 145, IQR 114.5–1176.5 vs. median 123, IQR 100.5–141.5, *P* = 0.04). These findings indicate that baseline patient characteristics exert limited influence on the development of post-CPB ALI. Conversely, both operative duration and CPB duration were closely correlated with ALI. Comparing clinical outcomes between coronary heart disease patients with CPB-associated ALI and NALI, postoperative PaO_2_/FiO_2_ ratios were significantly lower in ALI patients (170.58 ± 48.048 vs. Median 265.95, IQR 209.75-349.25, *P* < 0.001), and ICU stay duration (median 44.05, IQR 24.31–113.22 vs. median 19.12, IQR 14.02–23.17, *P* < 0.001), ventilator support duration (median 44.5, IQR 21.25–71.5 vs. median 15.5, IQR 12.25–20.75, *P* < 0.001), and longer hospital stay (median 18, IQR 15.5–25.5 vs. median 14, IQR 12.25–20.75, *P* = 0.015).

**Table 1:** Demographic and clinical characteristics of two groups.

Characteristics	Total(n=53)	ALI(n=21)	NALI(n=32)	*P* value
Preoperative factors				
Age (years)	56.17 ± 10.59	59.1 ± 8.25	54.25 ± 11.6	0.104
Male (n)	34 (64.2)	16 (76.2)	18 (56.3)	0.139
BMI, kg/m^2^	24.86 ± 3.12	24.69 ± 3.34	24.97 ± 3.02	0.753
Hypertension (n, %)	30 (56.6)	15 (71.4)	15 (46.9)	0.078
Diabetes (n, %)	6 (11.3)	3 (14.3)	3 (9.4)	0.671
Hyperlipidemia (n, %)	12 (22.6)	5 (23.8)	7 (21.9)	1
Cerebrovascular disease (n, %)	7 (13.2)	4 (19)	3 (9.4)	0.415
SPO_2_	96.65 (95.83, 97.6)	96.2 (95.25, 97.2)	96.7 (96, 97.7)	0.222
LVEF (%)	62 (56, 52.5)	61 (56.5, 65)	63.5 (55.5, 66)	0.444
Operative factors				
Operate time (mins)	294.55 ± 77.32	334.21 ± 89.36	268.52 ± 55.78	0.002*
CPB time (mins)	128 (105, 156.6)	145 (114.5, 176.5)	123 (100.5, 141.5)	0.04*
ACC time (mins)	82 (70, 104)	97.84 ± 34.59	78 (69.5, 99.75)	0.115
Postoperative factors				
PaO_2_/FiO_2_	215 (187, 300.95)	170.58 ± 48.08	265.95 (209.75, 349.25)	<0.001
Clinical Outcomes				
ICU time (hours)	37.78 ± 40.31	64.91 ± 52.53	19.4 ± 7.62	0.001*
Vt time (hours)	19.5 (14.25, 26)	44.5 (21.25, 71.5)	15.5 (12.25, 20.75)	0.001*
Hospital stays (days)	17 (113, 23)	18 (15.5, 25.5)	14 (12.25, 20.75)	0.015*

### 4.2 Gut microbiota analysis

#### 4.2.1 Gut microbiota community characteristics in two patient groups

**Figure 1:**
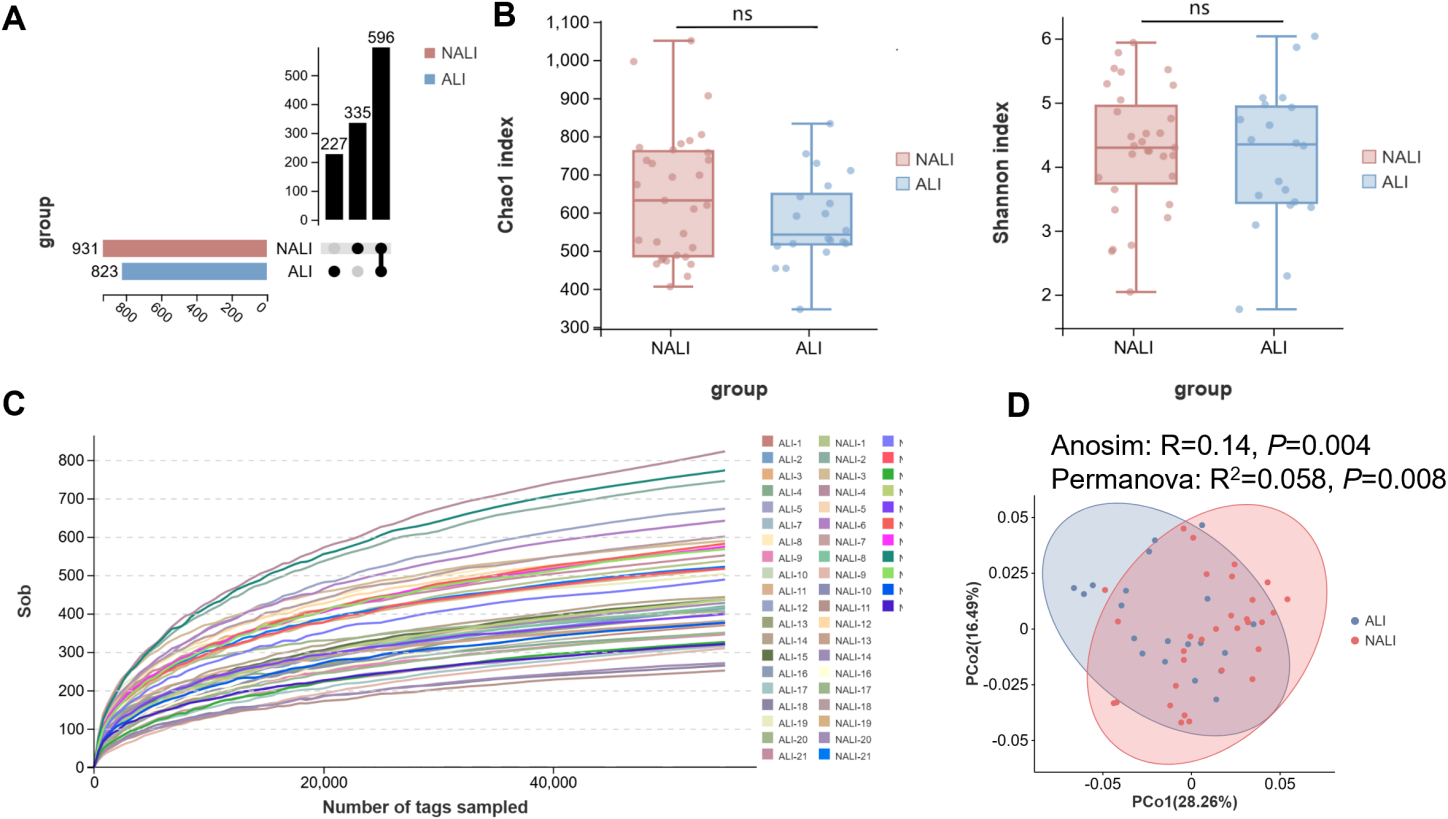
Microbial differential analysis between ALI and NALI groups. **(A)** Upset plot showing the number of OTUs per group. **(B)** No significant difference in α diversity between ALI and NALI populations, as estimated by Chao 1 index and Shannon index. **(C)** Sparcity curve analysis. **(D)** β diversity analysis based on PCoA plot.

Sequencing analysis was performed on fecal samples from all enrolled patients. Across all specimens, a total of 1,754 OTUs were observed, comprising 931 OTUs in the NALI group and 823 OTUs in the ALI group. Shared OTUs between the two groups numbered 596, with 335 OTUs specific to NALI and 227 OTUs specific to ALI (**Figure 1A**). These findings indicate that patients without postoperative ALI possessed a richer microbiota compared to those with ALI. α-diversity reflects the state of diversity within the gut microbiome, typically assessed through species richness and evenness. Sob, Chao1, and Shannon are common indices for evaluating α-diversity, where Sob and Chao1 reflect species richness, while Shannon measures the evenness of species distribution. In α-diversity analysis, no significant differences were observed between groups for the Chao1 and Simpson indices (*P* > 0.05, **Figure 1B**). This indicates no significant disparity in gut microbial community richness or diversity between groups. To minimize bias due to uneven sequencing Depth, we diluted all samples to [55,000] sequences per sample (i.e., Rarefaction Depth) for subsequent diversity analyses. Sparcity curve analysis revealed a plateauing trend with increasing reads, confirming an adequate number of individual samples (**Figure 1C**). Furthermore, the OTU-based PCoA evaluation, based on weighted UniFrac, clearly demonstrated overlapping yet distinct microbial community structures between groups (Anosim: R = 0.14, *P* = 0.004; Permanova: R^2^ = 0.058, *P* = 0.008).

via β-diversity PCoA analysis (**Figure 1D**). Although the observed effect size was relatively small, the PERMANOVA test revealed highly significant between-group differences (pseudo-F = 3.0267), indicating that this grouping factor is a determining factor in shaping community structure. This indicates differences in microbial community composition between the two patient cohorts.

#### 4.2.2 Microbial community identification and species distribution

**Figure 2:**
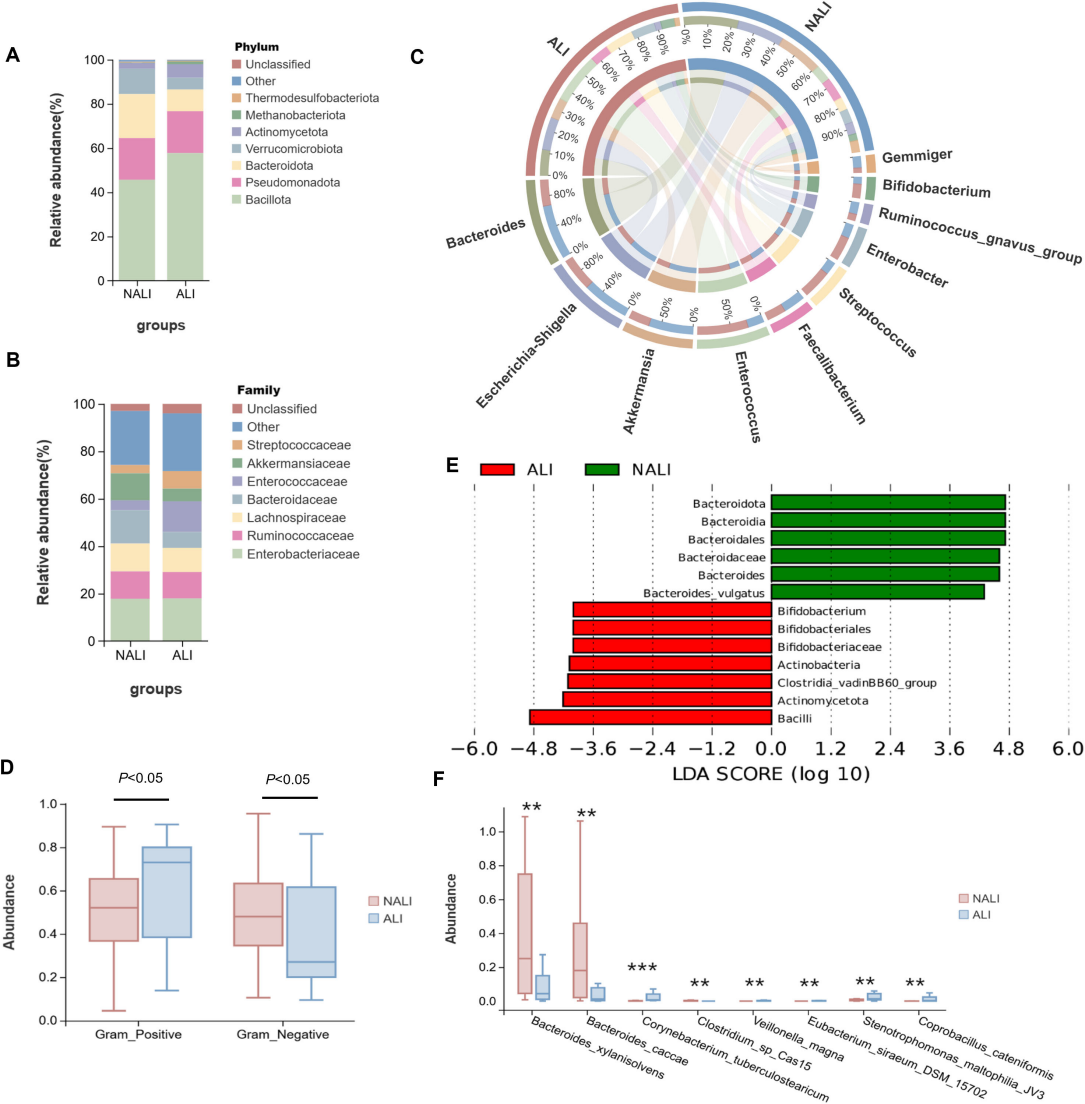
Microbial composition and abundance in NALI and ALI groups. Subgroup analysis of gut microbiota composition at phylum **(A)**, family **(B)**, and genus **(C)** levels. **(D)** Statistically significant bacterial phenotypic functions in the NALI and ALI groups based on BugBase prediction analysis. **(E)** LEfSe used to identify essential differences in bacterial abundance (genus to species level) between the CPB-ALI and CPB-NALI groups. Only taxa with a significant LDA threshold value > 2 are shown. **(F)** Abundance of the top 8 most differentially abundant species based on Wilcoxon rank-sum test. **p < 0.01, ***p < 0.001.

Significant differences in gut microbiota composition were observed between the two groups. At the phylum level, compared with NALI patients, the phylum Bacillota was increased in ALI patients, while the phyla Bacteroidetes and Actinomycetota were reduced (**Figure 2A**). At the family level, *Enterococcaceae* showed no significant difference between ALI and NALI patients, whereas *Streptococcaceae* increased and *Bacteroidaceae* and *Akkermansiaceae* decreased in ALI patients (**Figure 2B**). At the genus level, *Bacteroides*, *Escherichia-Shigella*, and *Akkermansia* were more abundant in NALI patients, whereas *Enterococcaceae* was more abundant in ALI patients (**Figure 2C**). The overall trend indicates a reduction in beneficial gut microbiota and an increase in harmful microbiota in the ALI group. Subgroup analysis indicated Gram-positive bacteria predominated in the NALI group, while Gram-negative bacteria were predominant in the ALI group (**Figure 2D**). Notably, *Bacteroides* was the dominant genus in the NALI group. In contrast, the ALI group exhibited higher enrichment of *Corynebacterium*, a common pro-inflammatory opportunistic pathogen. Furthermore, LefSe analysis was conducted to identify dominant bacterial taxa within the ALI and NALI groups. Results revealed higher abundances of *Bacilli* and *Actinomycetota* in the ALI group, whereas *Bacteroidota* dominated the NALI group (**Figure 2E**). Finally, univariate analysis of variance was employed to examine the abundance of the top eight distinct species across the ALI and NALI groups (**Figure 2F**).

#### 4.2.3 Functional analysis of microbiome genes and correlation with clinical characteristics

**Figure 3:**
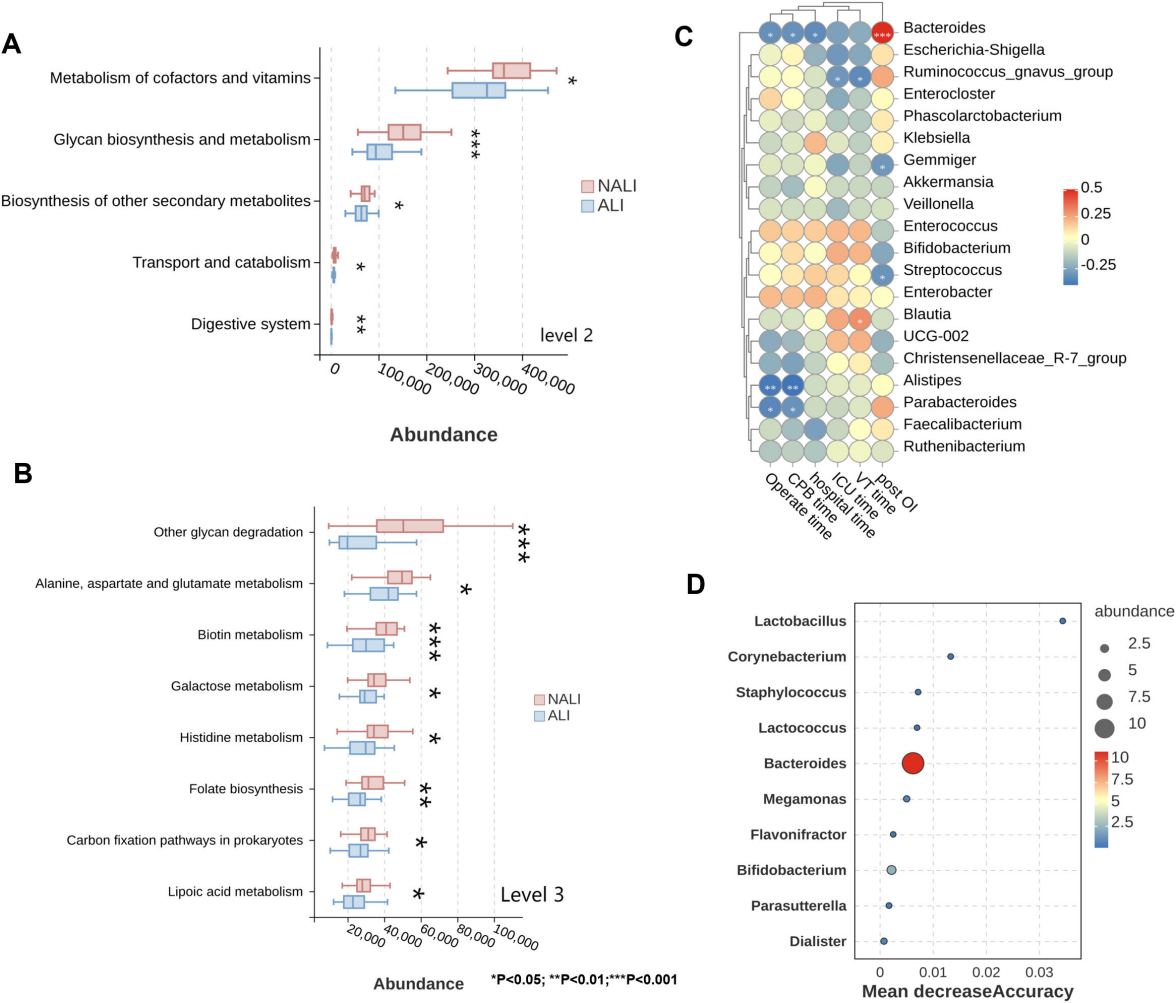
Microbial gene function and clinical correlations. **(A, B)** Functional annotations at levels 2 and 3 (Wilcoxon test). **(C)** Spearman correlation heatmap between microbiota and clinical indicators. **(D)** Top 10 biomarker bacteria by Mean Decrease Accuracy. *p < 0.05, **p < 0.01, ***p < 0.001. VT, Ventilator time; Post OI, Postoperative oxygenation index after CPB.

Furthermore, PICRUSt2 was employed for second- and third-level analysis of 16s rRNA sequencing data. The wilcoxon signed-rank test was employed to compare functional differences in the microbiota between the two groups of ALI patients. At the second level, we screened the top five significantly differentially annotated functions, revealing that patient microbiota distribution was significantly associated with metabolism of cofactors and vitamins, glycan biosynthesis and metabolism, biosynthesis of other secondary metabolites, transport and catabolism, and digestive system (**Figure 3A**). At the third level, we screened the top seven significantly enriched differential functions. Compared with ALI patients, NALI patients exhibited gut microbiota abundance closely associated with alanine, aspartate and glutamate metabolism, biotin metabolism, galactose metabolism, histidine metabolism, folate biosynthesis, carbon fixation pathways in prokaryotes; and lipoic acid metabolism (**Figure 3B**). Subsequently, to investigate the relationship between gut microbiota and various clinical characteristics (such as PaO_2_/FiO_2_, VT, hospital stay, CPB duration, and operating time), spearman correlation analysis was performed. Results indicated that gut microbiota differences may correlate with these clinical features. Bacteroides exhibited negative correlations with multiple clinical parameters, including operative time, CPB duration, and hospital stay, while showing a positive correlation with postoperative PaO_2_/FiO_2_. *Alistipes* and *Parabacteroides* demonstrated negative correlations with operative time and CPB duration (**Figure 3C**).

By applying random forest regression to the relative abundance values of these key microbial communities, the top ten bacterial categories serving as biomarkers were identified (**Figure 3D**). ROC curve analysis revealed the predictive capability of these key microbial communities for ALI (**Table 2**). Bacteroides (AUC 0.723, 95% CI: 0.574–0.871), Corynebacterium (AUC 0.798, 95% CI: 0.679–0.918), and Lactobacillus (AUC 0.859, 95% CI: 0.76–0.958) demonstrated predictive value for the disease (**Supplementary Figure 1**). To improve model transparency, the random forest classifier was evaluated using out-of-bag (OOB) estimation. The OOB error rate was 18.9%, consistent with internal stability of the model. Given the modest sample size and high dimensionality of microbiome data, we explicitly acknowledge the possibility of overfitting, and therefore interpret the random-forest results as exploratory biomarkers rather than definitive diagnostic indicators (**Supplementary Figure 2**).

**Table 2:** The ability of specific microbiome biomarker in predicting ALI.

Factor	AUC	95% CI (AUC)	Best thresholds (specificities, sensitivities)
Bacteroides	0.723	0.574~0.871	5.3271 (0.75, 0.742)
Bifidobacterium	0.689	0.533~0.844	3.24605 (0.45, 0.935)
Dialister	0.634	0.474~0.794	0.291 (0.95, 0.323)
Megamonas	0.667	0.517~0.817	0.0048 (0.85, 0.516)
Parasutterella	0.699	0.546~0.852	0.00195 (0.4, 0.968)
Staphylococcus	0.731	0.581~0.88	0.03285 (0.55, 0.903)
Corynebacterium	0.798	0.679~0.918	0.00485 (0.9, 0.581)
Lactobacillus	0.859	0.76~0.958	0.0024 (1, 0.581)
Flavonifractor	0.71	0.565~0.855	0.03155 (0.7, 0.71)
Lactococcus	0.717	0.578~0.856	0.00055 (0.85, 0.645)

### 4.3 Differences in metabolites between ALI and NALI

**Figure 4:**
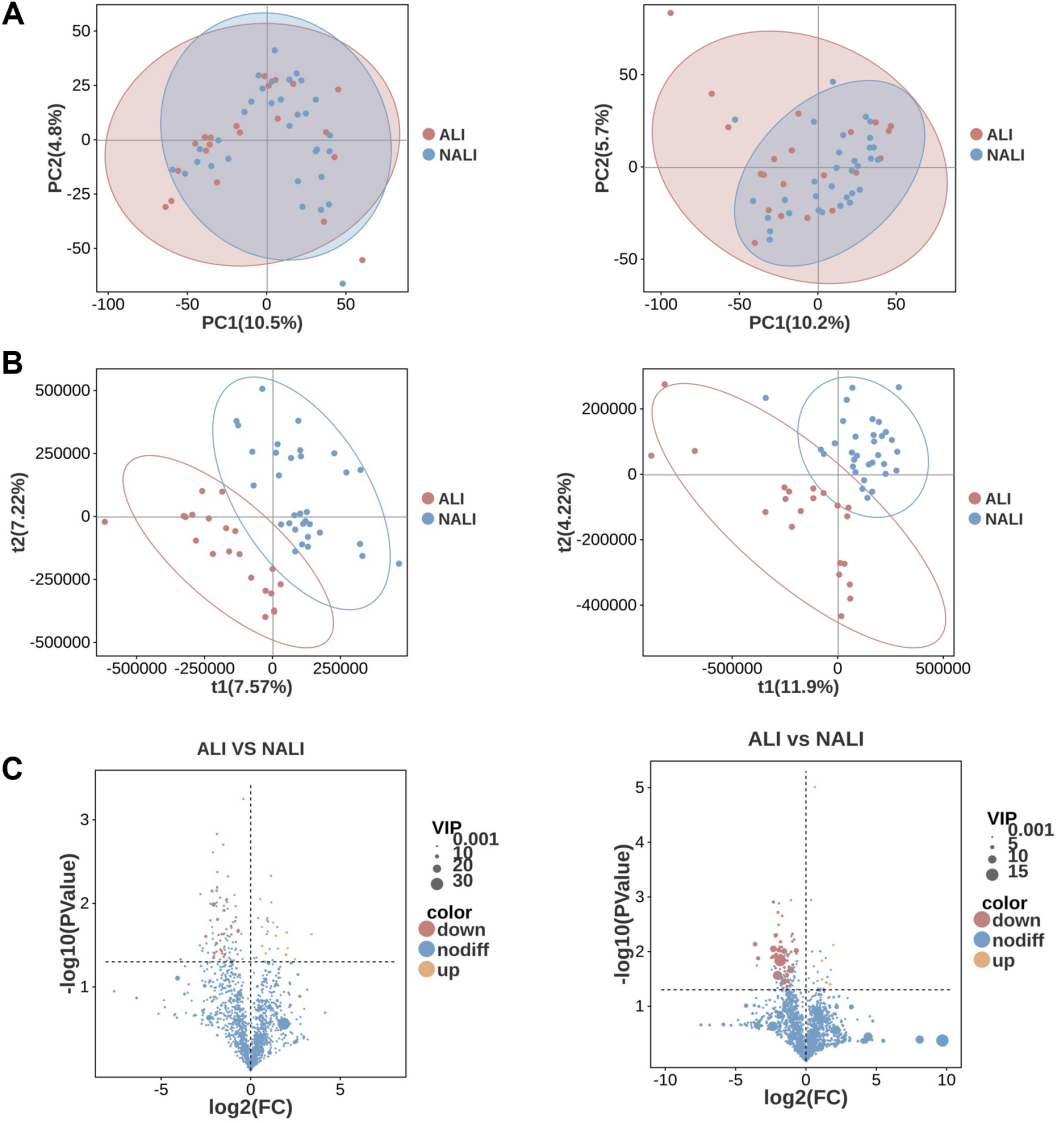
PCA and PLS-DA scoring plots and volcano plot of metabolites. **(A)** PCA in NEG and POS modes. **(B)** PLS-DA scoring plot. **(C)** Volcano plot: x-axis, log2FC; y-axis, -log10(p-value); red/blue indicate up/down-regulated metabolites; dot size represents VIP.

Subsequently, we conducted non-targeted metabolomic analysis on fecal samples from ALI and NALI patients. Scoring plots generated using PCA models revealed effective separation of the first three principal components between the two groups. PCA analysis revealed markedly distinct metabolic profiles between patient groups in both positive and negative ion modes, though separation was not significant. Subsequently, PLS-DA modeling demonstrated pronounced clustering of metabolic signatures between groups, indicating clear separation (**Figure 4**).

**Figure 5:**
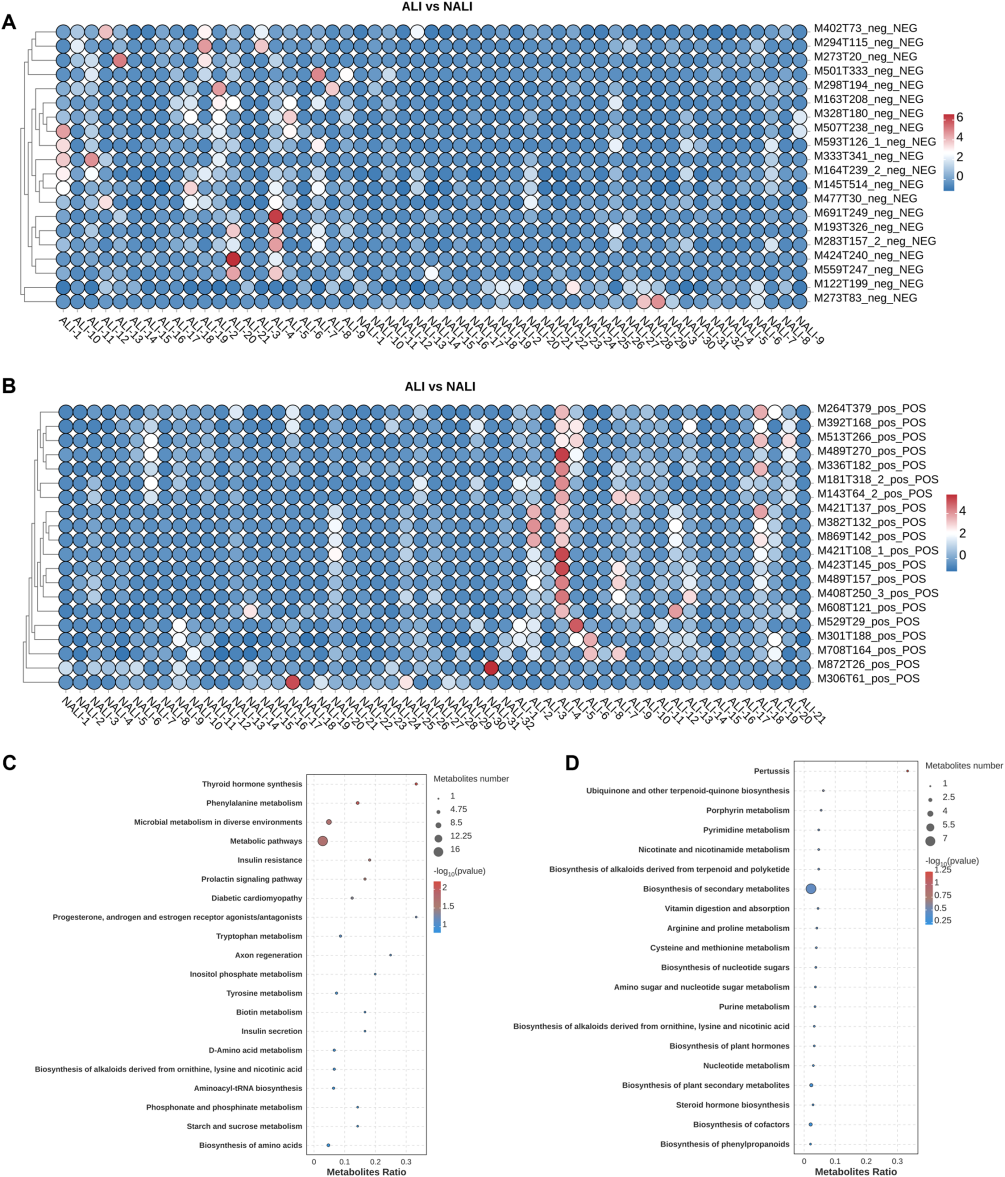
Differential metabolites and KEGG pathways. **(A, B)** Heatmaps in NEG and POS modes. The colors from blue to red indicate the relative contents of the metabolites in the two groups. **(C, D)** Top 20 KEGG pathways in NEG and POS modes.

A total of 1,886 POS mode metabolites and 1,507 NEG mode metabolites were identified in ALI and NALI patients respectively (n=53) (**Supplementary Table S1**). Hierarchical clustering was performed on the differentially expressed metabolite patterns, with heatmaps illustrating the overall profiles of distinct metabolic signatures (**Figures 5A, B**). Based on VIP >1 in the loading plot, FC≥1 or FC ≤1, and P < 0.05, 130 differentially accumulated metabolites were identified. Among these, 21 metabolites were significantly enriched in the ALI group, while the remaining 109 metabolites were depleted, suggesting potentially diminished metabolic activity in these patients (**Supplementary Table S2**). Consequently, these metabolites were selected as reference points for subsequent analysis. Furthermore, metabolic enrichment and pathway analysis based on KEGG annotation mapped these ALI-associated metabolites to their biochemical pathways. They were found to predominantly participate in Global and overview maps, including Microbial metabolism in diverse environments and Metabolic pathways. Differentially expressed metabolites also appeared closely associated with amino acid metabolism, encompassing: phenylalanine metabolism, tryptophan metabolism, tyrosine metabolism, lysine biosynthesis, lysine degradation, glycine, serine and threonine metabolism, alanine, aspartate and glutamate metabolism (**Figures 5C, D**).

### 4.4 Cross-correlation analysis between the microbiota and metabolites

**Figure 6:**
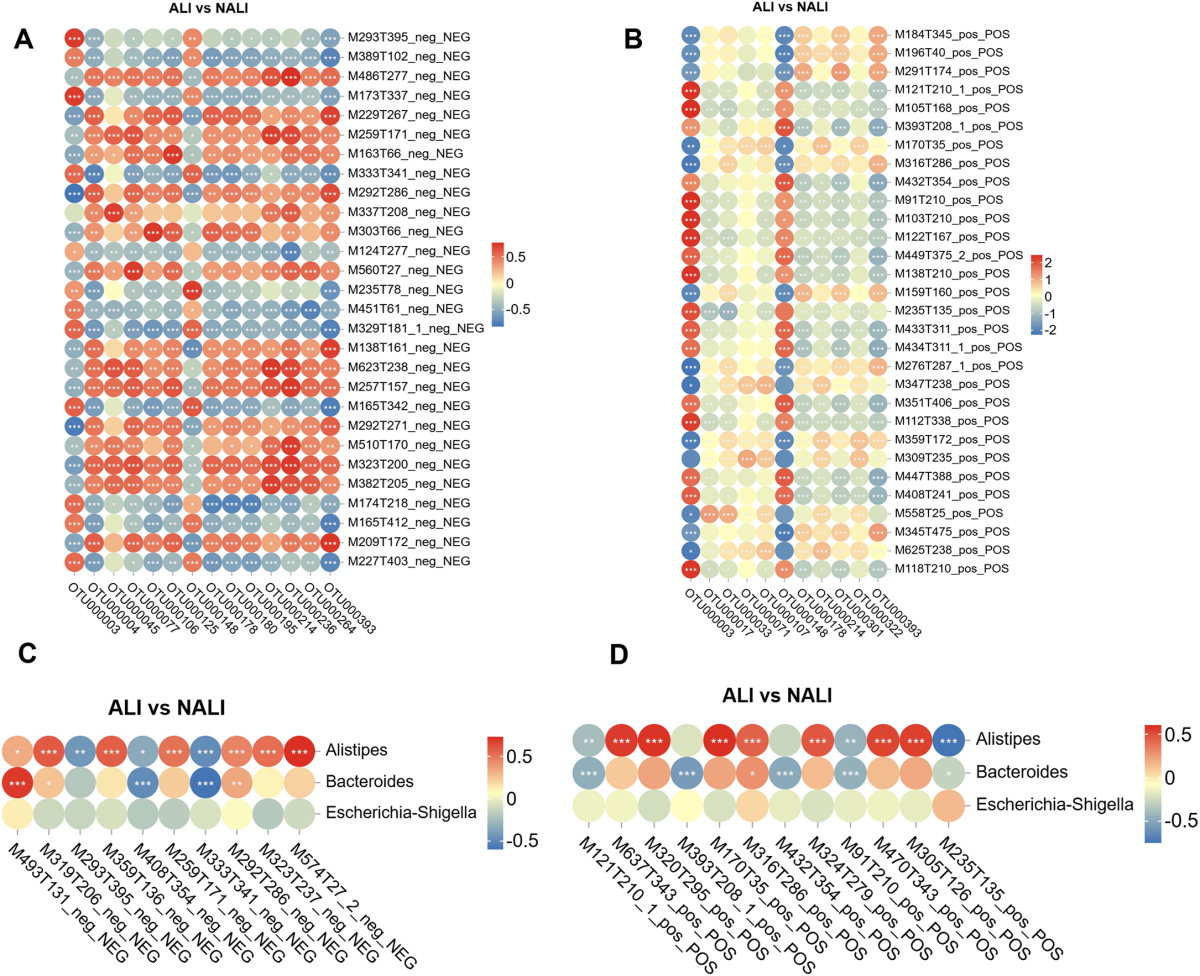
Microbiota-metabolite correlations. **(A, B)** Spearman heatmaps of top 30 OTUs and metabolites. **(C, D)** Correlation heatmaps of three genera and metabolites in NEG and POS modes. Red: positive correlation; blue: negative correlation. *p < 0.05, **p < 0.01, ***p < 0.001.

To explore the functional relationships between the differential gut microbiota and differentially accumulated fecal metabolites in ALI and NALI patients, we conducted correlation analyses based on Spearman’s correlation coefficients. Microorganisms and metabolites annotated at the genus level that exhibited statistically significant differences based on correlation coefficients were included in the analysis. Heatmaps were generated to illustrate the strength and polarity of correlations. Based on correlation coefficients, the top 30 statistically significant OTUs and differentially accumulated metabolites were displayed, with correlation heatmaps plotting gut microbiota and metabolites (**Figures 6A, B**). This demonstrates that gut metabolites are closely associated with the microbial community composition of ALI patients. Furthermore, prior findings from microbiome composition studies indicated that *Bacteroides*, *Escherichia-Shigella*, and *Alistipes* genera were associated with lung injury occurrence. All pairwise microbiome–metabolite correlations were calculated using the Spearman method. To control for multiple comparisons, FDR correction was applied using the Benjamini–Hochberg procedure, and only associations with FDR-adjusted *P* < 0.05 were considered significant. In addition to heatmap visualization, a network diagram illustrating the top 50 significant microbe–metabolite associations. (**Supplementary Figure 3**). Subsequently, we conducted cross-correlation analyses between these three microbial groups and differentially accumulated metabolites, presenting metabolites matched to MS2 names (**Figures 6C, D**). Metabolite IDs correspond to matched MS2 names (**Supplementary Table S3**).

## 5 Discussion

In this study, we investigated the gut microbiological and metabolic characteristics of patients with ALI following CPB surgery, compared with those who did not develop ALI. Our aim was to explore correlations between gut microbiota, metabolic products, and clinical indicators in post-CPB patients, with the objective of identifying potential biomarkers and providing evidence for targeted prevention and treatment of post-CPB ALI. Our findings revealed significant differences in gut microbial composition and diversity between the two groups, alongside variations in microbial community distribution at multiple levels. Specifically, we observed elevated levels of pro-inflammatory gut microbes and reduced levels of anti-inflammatory gut microbes in CPB-associated ALI patients. Furthermore, ALI patients exhibited distinct metabolic profiles compared to NALI patients, with metabolites showing correlations with gut microbiota.

Regarding gut microbiota, we observed no differences in diversity or richness between ALI and NALI patients. This community similarity likely stems from shared core species essential for maintaining gut diversity despite differing health states. β-diversity analysis revealed significant differences in microbial community composition between groups (Anosim: R = 0.14, *P* = 0.004; Permanova: R^2^ = 0.058, *P* = 0.008). This suggests that microbial composition, rather than overall diversity, may be the key factor influencing the development of ALI.

We observed significant differences in gut microbiota composition between the two groups. At the phylum level, compared with patients in the NALI group, the phylum *Bacillota* was increased in ALI patients, while the phyla *Bacteroidetes* and *Actinomycetota* were reduced (**Figure 2A**). *Bacillota* and *Bacteroidetes* represent two major bacterial phyla within the gut microbiota. An elevated Bacillota/Bacteroidetes ratio has been recognized in multiple studies as a marker of gut dysbiosis, potentially associated with heightened systemic inflammatory responses (22, 23). In our study, the *Bacillota*/*Bacteroidetes* ratio was reduced in ALI patients. This mirrors findings by Jiang et al., who observed that in infants developing ALI after CPB, preoperative *Bacillota* abundance showed no difference compared to healthy volunteers or infants without ALI, whereas *Bacteroidetes* abundance was markedly decreased. Modulating the *Bacillota*/*Bacteroidetes* ratio may mitigate ALI (13). In an acute pancreatitis-associated ALI model, dietary fiber (pectin) regulated the gut *Bacillota*/*Bacteroidetes* ratio, thereby influencing immune balance and alleviating lung injury (24). At the family and genus levels, the *Streptococcaceae* and *Enterococcaceae* families were enriched in the ALI group, while the *Bacteroidaceae* and *Akkermansiaceae* families were reduced. As opportunistic pathogens, increased abundance of *Streptococcaceae* and *Enterococcaceae* may correlate with exacerbated pulmonary inflammation and poor prognosis (25). Notably, *Enterococcaceae* in ICU patients showed significant association with increased hospital-acquired infection and mortality risk (26). Conversely, *Bacteroides* and *Akkermansia* are generally recognized for their anti-inflammatory and gut barrier-protective functions; their reduction may compromise mucosal immunity, promote bacterial and metabolite translocation, and thereby exacerbate damage to distal organs such as the lungs (27). For instance, recent studies reveal a negative correlation between *Akkermansia* and inflammatory mediators TNF-α, IL-1β, and IL-6, suggesting its potential protective role in suppressing systemic inflammation (28). In summary, the overall trend in microbial communities indicates a reduction in beneficial bacteria and an increase in harmful bacteria within the gut microbiota of patients with ALI.

Through functional prediction analysis using PICRUSt2, we identified significant alterations in the microbiota of ALI patients across multiple metabolic pathways, particularly those involving amino acid metabolism (e.g., alanine, aspartate, and glutamate metabolism) and vitamin synthesis pathways (e.g., biotin and folate metabolism). These pathway alterations may influence host immune cell function, oxidative stress levels, and energy metabolism, thereby contributing to the pathogenesis of ALI (29). For instance, tryptophan metabolites regulate immune cell differentiation and function via the aryl hydrocarbon receptor (AhR) pathway, thereby influencing pulmonary inflammation severity (30); whereas folate and biotin deficiencies may correlate with impaired epithelial repair and dysregulated immune modulation (31). Spearman correlation analysis revealed associations between gut microbiota community differences and clinical features of ALI. In this study, the relative abundance of Bacteroides showed a negative correlation with operating time, CPB duration, and hospital stay, while exhibiting a positive correlation with postoperative PO_2_/FiO_2_. *Alistipes* and *Parabacteroides* exhibited negative correlations with both operative duration and CPB time. Random forest modeling further identified key bacterial genera—*Bacteroides*, *Corynebacterium*, and Lactobacillus—as potential biomarkers. These demonstrated high predictive accuracy for post-CPB ALI, with AUC> 0.7.

Regarding metabolomics, numerous studies have demonstrated that alterations in the metabolite profile are associated with various diseases, such as Parkinson’s disease (32), ischaemic stroke (33), and depression (34), yet few studies have focused on ALI. In our study, non-targeted metabolomics analysis revealed distinct metabolic signatures in ALI patients compared to NALI patients. Among ALI patients, 109 metabolites were significantly depleted, while only 21 were enriched, suggesting potentially widespread impairment in overall metabolic activity. PLS-DA analysis demonstrated clear separation between the two groups. Through KEGG enrichment analysis, we identified metabolites primarily involved in amino acid metabolism pathways, such as phenylalanine, tryptophan, and tyrosine metabolism. These pathways are closely associated with inflammatory regulation, neuroendocrine function, and immune responses (35, 36).

Notably, these findings show high concordance with gut microbiota functional predictions, suggesting that alterations in microbial metabolic functions may serve as a bridging mechanism in the pathogenesis of ALI. For instance, tryptophan derivatives (such as indole compounds) can modulate macrophage and T-cell responses via the AhR signaling pathway, thereby influencing pulmonary inflammatory processes (37, 38); certain lipid metabolites may directly participate in the resolution or exacerbation of inflammation.

Combined analysis of key bacterial genera *Alistipes*, *Bacteroides*, and the focus group *Escherichia-Shigella* with the metabolome revealed significant correlations between these genera and differential metabolites, such as certain amino acid derivatives and short-chain fatty acids. In our study, Spearman correlation analysis demonstrated certain associations between the gut microbiota and fecal metabolites. While metabolomics analysis alone cannot establish definitive causality, we identified several correlations between the two. Notably, we observed that Alistipes abundance correlated with elevated levels of Sulfobacin b, Chloroquine, Acetylpodocarpic acid anhydride, Linustatin, and Zeranol. The abundance of *Escherichia-Shigella* also exhibited correlations with metabolites, though these results were not statistically significant. This contrasts with literature reports suggesting Escherichia-Shigella serves as a key microbial indicator for predicting postoperative acute ALI. This discrepancy may stem from widespread distribution abnormalities observed postoperatively compared to preoperative states. These findings indicate that abnormal metabolic activity during the development of CPB-associated ALI may be closely linked to gut microbiota function. This further supports the regulatory role of the gut-lung axis in CPB-induced ALI. This discovery offers potential for developing early warning strategies based on microbiota and metabolites in the future.

Several of the observed microbe–metabolite associations have established biological relevance within the gut–lung axis. Decreased abundance of beneficial taxa was accompanied by reduced short-chain fatty acid (SCFA)–producing pathways, which may impair GPR41/43-mediated anti-inflammatory signaling (39). Meanwhile, altered tryptophan metabolism, particularly reduced levels of indole derivatives, may diminish aryl hydrocarbon receptor (AhR) activation, thereby weakening mucosal immune homeostasis (30). Increased succinate and other pro-inflammatory metabolites suggest activation of neutrophil-driven inflammatory pathways, consistent with the pathophysiology of acute lung injury (16, 40). These findings support a mechanistic link between microbiome dysbiosis, metabolite imbalance, and pulmonary immune activation following CPB. Based on current evidence and our findings, a conceptual mechanistic framework can be proposed. CPB triggers systemic inflammation and intestinal hypoperfusion, leading to increased gut permeability and dysbiosis. Loss of SCFA-producing commensals reduces epithelial barrier integrity and blunts anti-inflammatory signaling through GPR41/43. Altered tryptophan metabolism decreases AhR activation, impairing mucosal immune regulation. Concurrent increases in succinate and other pro-inflammatory metabolites may amplify IL-1β and neutrophil-driven pathways. These gut-derived inflammatory signals can reach the lungs through hematogenous routes, priming alveolar macrophages and neutrophils and promoting the development of ALI.

This study also presents several limitations. Firstly, the sample size was small (n=53) and derived from a single center, failing to account for the potential influence of differing environments, diets, surgical procedures, and other factors on the gut microbiota and metabolites. This may constrain the generalizability and extrapolation of the findings. Secondly, Preoperative fecal samples were not collected due to clinical constraints, including fasting protocols, bowel preparation procedures, and the frequent absence of spontaneous defecation before surgery, which limited our ability to characterize dynamic microbiome trajectories. Thirdly, this study was designed as an exploratory pilot cohort intended to generate hypotheses regarding the gut–lung axis in CPB-associated ALI. Because available evidence on postoperative microbiome dynamics after CPB remains limited, we performed a *post hoc* feasibility and power assessment. Based on prior literature, effect sizes for β-diversity differences between clinical phenotypes often range from R² = 0.05–0.15. We acknowledge that the sample size is not powered for small effect sizes or extensive multivariable modeling; therefore, all findings should be interpreted as preliminary and hypothesis-generating. Furthermore, owing to the observational study design, the observed results were not validated in animal models, precluding the establishment of a causal relationship between microbiota/metabolite alterations and ALI. Finally, only fecal samples were assessed; plasma metabolomics, inflammatory cytokines, and epithelial barrier markers were not collected, preventing direct assessment of systemic gut–lung signaling. Future work should expand validation through multicenter, large-scale cohort studies, combined with animal models (such as microbiota transplantation experiments) and *in vitro* mechanistic research to elucidate the specific roles and mechanisms of particular microbiota and metabolites in ALI pathogenesis.

In summary, this study systematically reveals that patients with acute lung injury following cardiopulmonary bypass surgery exhibit significant dysbiosis of the gut microbiota and metabolic dysfunction, both of which are closely interrelated and associated with adverse clinical outcomes. These findings not only deepen our understanding of the pathogenesis of post-CPB ALI but also provide a theoretical basis and potential targets for future development of early diagnostic biomarkers and interventions based on microbiota and metabolites, such as probiotics, prebiotics, or metabolite supplements.

## 6 Conclusion

This study integrated 16S rRNA sequencing and non-targeted metabolomics to reveal significant alterations in gut microbial community structure and metabolic function in patients developing ALI after CPB cardiac surgery. Postoperative ALI patients exhibited marked gut microbiota dysbiosis and widespread metabolic disruption. Correlation analysis indicated significant associations between gut microbiota and metabolites, suggesting microbial regulation of gut-lung axis function via metabolic products. Random forest modeling identified *Bacteroides*, *Corynebacterium*, and *Lactobacillus* as potential biomarkers with predictive capacity for ALI (AUC > 0.7). Clinical correlation analysis revealed that ALI patients exhibited poorer postoperative oxygenation, longer mechanical ventilation duration, and extended hospital stays. Furthermore, significant correlations were observed between ALI incidence and both operative duration and CPB time, further underscoring the critical role of gut microbiota and metabolites in clinical outcomes. The findings of this study provide novel theoretical and experimental evidence for understanding the gut-lung axis mechanisms underlying ALI development after CPB. They also offer potential targets for future development of early diagnostic strategies and interventions (e.g., probiotics, metabolite supplements) based on microbiota and metabolites. However, this study remains a single-center, small-sample observational investigation. Its findings require validation through multicenter, large-scale studies, alongside animal experiments and mechanistic research to clarify causal relationships and specific pathways of action.

## Data Availability

The datasets presented in this study can be found in online repositories. China National Center for Bioinformation (GSA:CRA030433) that are publicly accessible at https://ngdc.cncb.ac.cn/gsa and OMIX (https://ngdc.cncb.ac.cn/omix: OMIX012022). The names of the repository/repositories and accession number(s) can be found in the article/**Supplementary Material**.
