# Supplementary material for: Study on the characteristics and correlation of fecal microbiota and metabolites in patients with acute lung injury after cardiopulmonary bypass based on 16S rRNA sequencing and non-targeted metabolomics analysis
Source: Front Immunol. 2026 Jan 12;16:1713650. doi: 10.3389/fimmu.2025.1713650 (PMC12832994; doi:10.3389/fimmu.2025.1713650)
Supplement: Supplementary file 1 [file Supplementaryfile1.docx]

**Supplementary Figure 1**


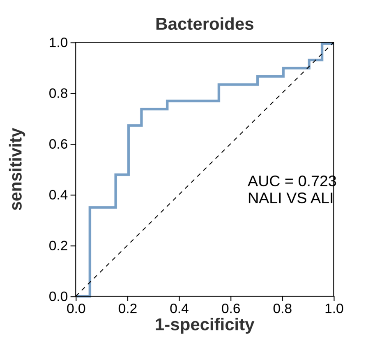

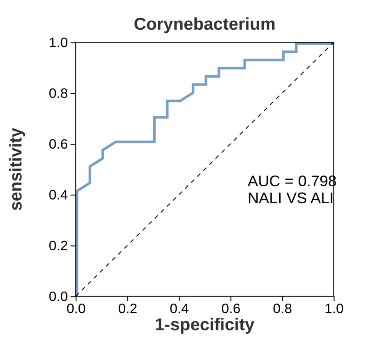

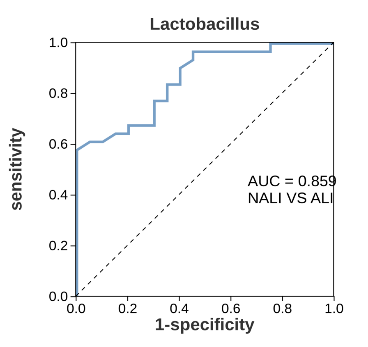


95% CI=0.574~0.871

95% CI=0.679~0.918

95% CI=0.76~0.958

**ROC curves**. ROC curves showed the ability of *Bacteroides*, *Corynebacterium*, and *Lactobacillus* biomarker in predicting CPB-ALI.

**Supplementary Figure 2
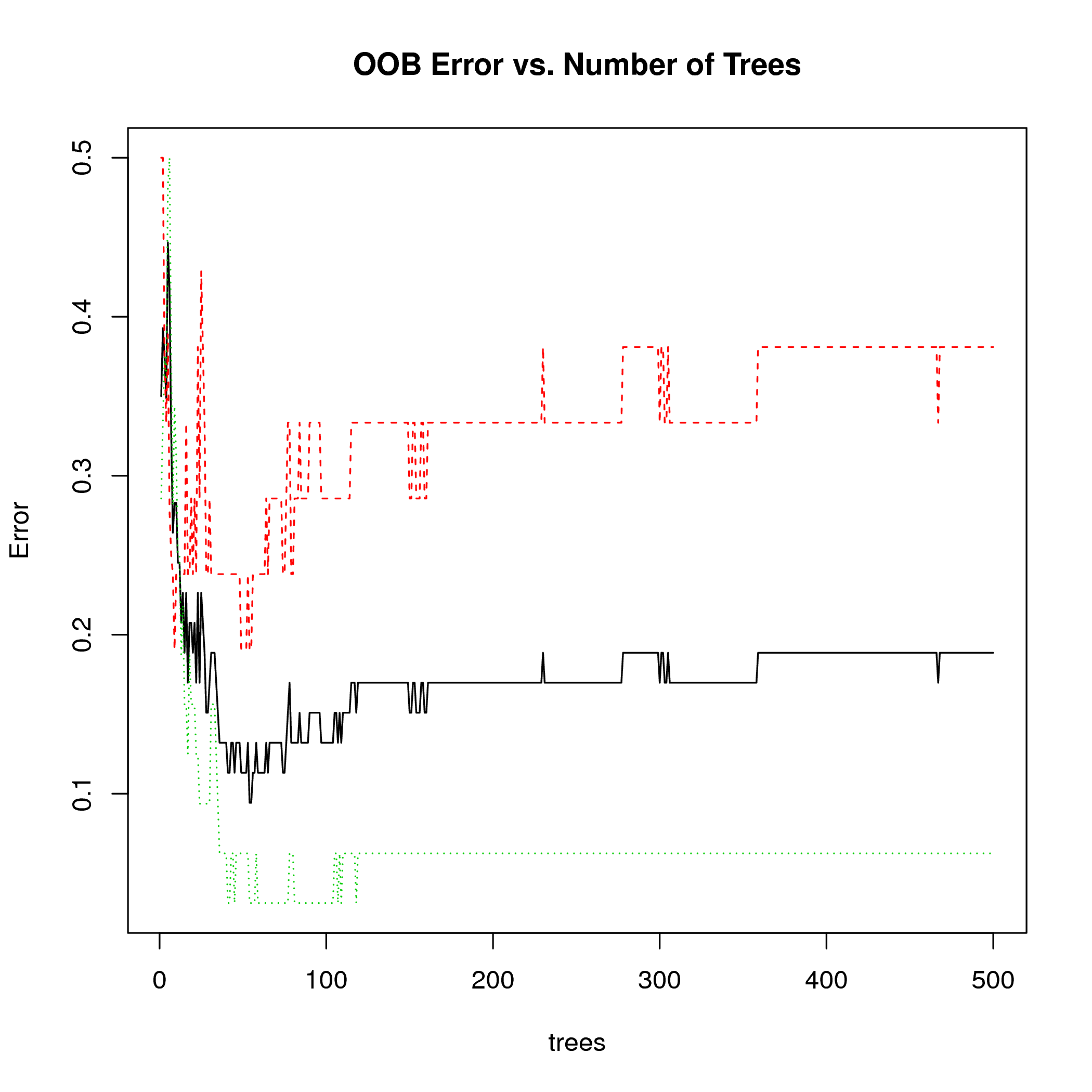
**

**OOB error rate graph in Genus level.**

**Supplementary Figure 3**


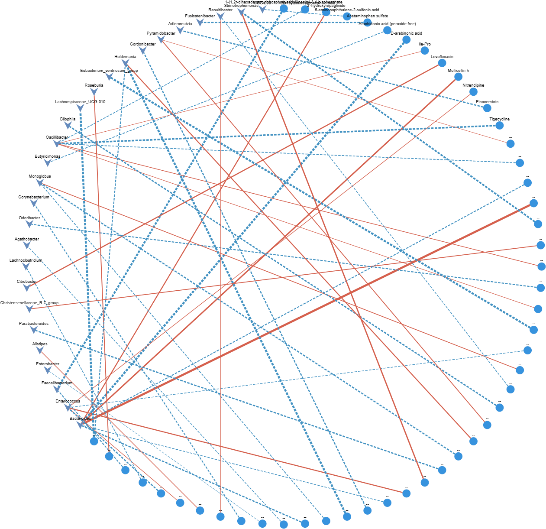

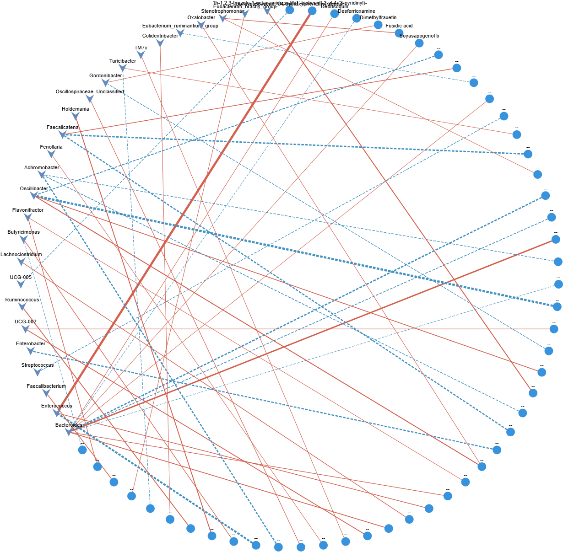


A

B

**The network diagram of microbiome–metabolite correlation.** (A) NEG modes (B) POS modes.
